# Alarm Fatigue and Missed Nursing Care Among Neonatal and Paediatric Intensive Care Nurses: A Cross‐Sectional Study

**DOI:** 10.1111/nicc.70649

**Published:** 2026-08-24

**Authors:** Buket Likos, Fatma Tanrıkulu

**Affiliations:** ^1^ Department of Fundamentals of Nursing Graduate Education Institute, Sakarya University of Applied Sciences Sakarya Turkey; ^2^ Department of Nursing, Faculty of Health Sciences Sakarya University of Applied Sciences Sakarya Turkey

**Keywords:** alarm fatigue, intensive care units, missed nursing care, neonatal, paediatric

## Abstract

**Background:**

Intensive care units (ICUs) are high‐technology environments where patient safety is monitored through various devices. However, the high frequency of alarms can lead to alarm fatigue, especially in neonatal and paediatric ICUs due to rapid physiological fluctuations. This condition may decrease nurses' attention and lead to missed nursing care.

**Aims:**

The primary aim of this study was to determine the levels of alarm fatigue and missed nursing care, along with the underlying reasons for missed care, among neonatal and paediatric intensive care nurses. The secondary aim was to examine the relationship between alarm fatigue levels and missed nursing care.

**Study Design:**

This descriptive, cross‐sectional correlational study was conducted in the neonatal and paediatric intensive care units of two public hospitals in Türkiye. Data were collected using self‐administered instruments, including the Nurse Characteristics Form, Alarm Fatigue Questionnaire and MISSCARE Survey—Paediatric Version.

**Results:**

The sample consisted of 175 neonatal and paediatric intensive care nurses. The mean alarm fatigue score was 15.46 ± 5.88. Labour and communication resources factors were prominent reasons for missed nursing care. A positive and statistically significant relationship was found between alarm fatigue and the reasons for missed nursing care, including labour (*r* = 0.243, *p* = 0.001), communication (*r* = 0.208, *p* = 0.006) and material resource factors (*r* = 0.268, *p* < 0.001).

**Conclusions:**

Effective alarm management strategies, adequate staffing and improved communication processes may help reduce missed nursing care and enhance patient safety in neonatal and paediatric intensive care units.

**Relevance to Clinical Practice:**

Reducing alarm fatigue in paediatric intensive care settings is essential. However, given the clinical vulnerability of paediatric patients, the life‐saving nature of these alarms mandates the provision of in‐service training on alarm management and the optimisation of patient‐specific alarm settings. Furthermore, to enhance the quality of care and optimise patient safety, it is recommended that institutional resources be improved and effective inter‐professional communication strategies be developed.

## Impact Statements

- What is known about the topic
  ○ In NICU and PICU settings, the high frequency of alarms due to rapid physiological fluctuations may reduce nurses' capacity to respond to alarms in a timely and effective manner.
  ○ Labour and workload‐related factors are among the most common determinants of missed nursing care.
- What this paper adds
  ○ Alarm fatigue in NICU and PICU settings is not solely an individual issue but is directly associated with missed nursing care.
  ○ As alarm fatigue increases, care omissions particularly in areas such as mobilisation, communication and continuity of care may also increase.
  ○ The findings highlight the need for holistic interventions targeting both alarm management and workforce planning.

## 1 Introduction

Intensive care units (ICUs) are structured environments designed to provide advanced medical and nursing care to patients with life‐threatening conditions and organ dysfunction [1]. In these units, an extensive range of technological equipment including monitors, ventilators, infusion pumps and feeding devices is utilised to stabilise vital parameters, support organ function and optimise the recovery process [2, 3]. However, the continuous auditory and visual stimuli generated by these devices can lead to a clinical desensitisation known as alarm fatigue among nurses, thereby potentially compromising patient safety [4, 5].

Alarm fatigue is a state of sensory overload and desensitisation towards alarms, resulting from clinicians' exposure to an excessive volume of alarm alerts [6, 7]. The sustained cognitive and physical effort required for alarm management, particularly under high‐workload conditions, can lead to the postponement or neglect of essential nursing interventions [8]. Research indicates that between 85% and 99% of alarms in ICUs are either false or clinically insignificant [7, 9]. Consequently, this phenomenon results in prolonged response times to genuine, life‐threatening alerts or, in some cases, their complete disregard. Ineffective alarm management and unsafe threshold settings trigger medical errors, delay the recovery process and pose significant life‐threatening risks [6, 10]. Furthermore, the American Association of Critical‐Care Nurses has reported that alarm fatigue has consistently compromised patient safety for over a decade. Therefore, mitigating the adverse effects of alarm fatigue remains a matter of critical contemporary importance [11].

In paediatric and neonatal intensive care units, alarm management is of paramount importance due to the total vulnerability of patients and the inherent complexity of their treatment processes [12, 13]. It is reported that paediatric nurses are exposed to between 150 and 400 alarms per shift, spending approximately 35% of their time solely responding to these alerts [14]. Excessive alarm noise not only leads to nurse fatigue but also adversely affects the neurodevelopment of infants and disrupts their sleep patterns. The high frequency of false alarms hinders nurses' clinical focus and subsequently diminishes the overall quality of care [15]. When coupled with high‐intensity workloads and staffing shortages, alarm fatigue facilitates delays in responding to critical alerts and predisposes the healthcare environment to systemic deteriorations in the quality of care [10].

Recent evidence has further clarified the mechanisms through which alarm fatigue may affect nursing care processes. A scoping review synthesising findings from 32 studies concluded that repeated exposure to frequent or non‐actionable alarms contributes to sensory overload, emotional strain and progressive desensitisation among healthcare professionals, resulting in delayed responses to clinically significant alarms and threats to patient safety [5]. Similarly, frequent intensive care alarms have been reported to increase nurses' workload, undermine trust in alarm systems and interfere with the delivery of safe patient care [16]. From a theoretical perspective, these consequences may contribute to cognitive overload and attentional fragmentation, reducing nurses' capacity to perform all required care activities and increasing the likelihood of missed nursing care. As attentional resources become depleted, nurses may be more likely to delay, omit or deprioritise necessary nursing interventions, thereby increasing the risk of missed nursing care [7, 17, 18]. Missed nursing care is defined as the partial or complete omission or delay of required care activities [19, 20, 21]. When forced to prioritise under an excessive alarm load, nurses may postpone interventions that are perceived as non‐vital yet remain critical for recovery, such as family education, psychological support, oral hygiene and nutritional assistance [22, 23].

In this context, examining the relationship between alarm fatigue and missed nursing care in neonatal and paediatric intensive care units is of strategic importance for both strengthening patient safety and ensuring the sustainability of care quality. Although the literature has separately demonstrated the adverse effects of alarm fatigue on patient safety and the negative impact of missed nursing care on care quality and patient outcomes, studies directly investigating the relationship between these two variables remain limited [8, 11, 12, 13]. Furthermore, in Türkiye, nursing care in neonatal and paediatric intensive care units is delivered within increasingly complex care environments characterised by advanced monitoring technologies, high patient acuity and growing workforce demands. Although monitoring systems that enable continuous patient surveillance are widely used in these settings, evidence regarding the extent of alarm fatigue and its potential impact on nursing care processes remains limited. In particular, the scarcity of studies examining the relationship between alarm fatigue and missed nursing care within the Turkish healthcare context highlights the need for further investigation. Therefore, this study aimed to address the effects of alarm fatigue on nursing care processes from a holistic perspective, thereby providing evidence‐based contributions to clinical practice and managerial strategies. The findings are expected to inform the development of alarm management policies, the optimisation of workload allocation and the establishment of care models that prioritise patient safety.

### 1.1 Aims

The primary aim of this study was to determine the levels of alarm fatigue, missed nursing care and the underlying reasons for missed care among nurses working in neonatal and paediatric intensive care units. The secondary aim was to examine the relationship between nurses' levels of alarm fatigue and missed nursing care.

### 1.2 Research Questions

- What are the levels of alarm fatigue among nurses working in neonatal and paediatric intensive care units?
- What types of missed nursing care are reported by nurses working in neonatal and paediatric intensive care units, and what are the underlying reasons?
- Is there a statistically significant relationship between the reasons for missed nursing care and the levels of alarm fatigue among nurses working in neonatal and paediatric intensive care units?

## 2 Design and Methods

### 2.1 Setting and Sample

This study was conducted using a descriptive and cross‐sectional correlational design. The research focussed on assessing alarm fatigue levels and missed nursing care among nurses practicing in neonatal and paediatric intensive care units. The study setting included the Neonatal Intensive Care Units (NICU) and Paediatric Intensive Care Units (PICU) of two major public hospitals in Türkiye. Hospital A (a tertiary training and research hospital) and Hospital B (a large‐scale city hospital) were selected for the study setting. These units provide advanced care for critically ill neonatal and paediatric patients requiring varying levels of monitoring and treatment. Hospital A had a NICU capacity of 40 incubators and a PICU capacity of 20 beds, whereas Hospital B had a NICU capacity of 48 incubators and a PICU capacity of 28 beds. A total of 179 nurses were actively employed in the relevant units, including 79 nurses in Hospital A (49 in NICU, 30 in PICU) and 100 nurses in Hospital B (60 in NICU, 40 in PICU). A total population sampling (census) approach was adopted, aiming to include all eligible nurses meeting the inclusion criteria during the study period.

A post hoc power analysis was conducted using G*Power 3.1.9.4 for the Pearson's correlation analyses. Based on a two‐tailed significance level of 0.05 and a sample size of 175, the achieved statistical power was calculated as 0.95 for the correlation between alarm fatigue and material resources (*r* = 0.268), 0.79 for communication (*r* = 0.208) and 0.90 for labour resources (*r* = 0.243).

The reporting of this study adhered to the Strengthening the Reporting of Observational Studies in Epidemiology (STROBE) guidelines to ensure methodological rigour and transparency.

### 2.2 Inclusion and Exclusion Criteria

The inclusion criteria were: (1) being a registered nurse in the NICU or PICU, (2) providing voluntary informed consent. Nurses were excluded if they were on long‐term leave (e.g., maternity or medical leave) or held purely administrative positions without direct bedside care responsibilities.

### 2.3 Data Collection Tools and Methods

Following formal ethics approval and institutional permissions, data collection was carried out between August and October 2025. Data were collected during face‐to‐face visits to the units. After being informed about the study objectives, voluntary participation and confidentiality, nurses who agreed to participate completed the questionnaires independently. Written informed consent was obtained from all nurses who agreed to participate. The completion of each data collection form took approximately 10–15 min.

#### 2.3.1 Nurse Characteristics Form

The form, developed by the researcher based on a review of the literature [12, 22], was designed to identify nurses' sociodemographic characteristics and professional attributes.

#### 2.3.2 Alarm Fatigue Questionnaire (AFQ)

Originally developed by Torabizadeh et al. [24] and validated in Turkish by Alan et al. [25], this 9‐item scale measures alarm‐related stress, desensitisation and response delays. Items are rated on a 5‐point Likert scale (0 = Never, 4 = Always), with higher scores indicating greater alarm fatigue. While the Turkish validation reported a Cronbach's alpha of 0.80, it was 0.768 in this study.

#### 2.3.3 MISSCARE Survey—Paediatric Version

This questionnaire was developed by Kalisch and Williams [26] to determine the frequency with which planned nursing care is missed in the paediatric patient population and to identify the potential reasons for this situation. It was subsequently expanded by Tubbs‐Cooley et al. [27] to encompass requirements specific to neonatal and paediatric care settings. The Turkish validity and reliability study of the instrument was conducted by Çalıkuşu İncekar et al. [28]. In the Turkish version, the intraclass correlation coefficient was reported as 0.75, and the Cronbach's alpha internal consistency coefficient was reported as α = 0.91. The scale consists of two sections. Section A comprises 29 items and assesses the frequency of each item. Section B examines the potential reasons for missed nursing care and consists of 16 items across three subdimensions (Material Resources, Communication and Labour Resources). Higher scores obtained from the subdimensions indicate a greater contribution of the corresponding factor to missed nursing care. In this study, Cronbach's alpha coefficients were 0.74 for Section A and 0.87 for Section B. For the Labour Resources, Communication and Material Resources subdimensions, Cronbach's alpha coefficients were 0.65, 0.79 and 0.72, respectively.

### 2.4 Statistical Analysis

The data were analysed using IBM SPSS Statistics 26, with the level of statistical significance set at *p* < 0.05. Frequency distributions (number, percentage) and descriptive statistics were used to evaluate the study data. The assumptions of normality were evaluated using skewness and kurtosis coefficients, descriptive statistics and the Shapiro–Wilk test. Although the Shapiro–Wilk test was significant for one variable, skewness and kurtosis values were within the acceptable range (±1), and the distributions were considered sufficiently normal for parametric analyses. Pearson's correlation analysis was applied to examine the relationships between the scale and its subdimension scores. In interpreting the correlation coefficient (*r*), values of 0.00–0.29 were considered weak, 0.30–0.69 moderate and 0.70–1.00 strong.

### 2.5 Ethics and Institutional Approvals

The study was conducted in accordance with the Declaration of Helsinki. Ethics approval was obtained from the Sakarya University of Applied Sciences Ethics Committee (Date: 7 May 2025; No: E‐26428519‐050.99‐172 770). Institutional permissions were secured from the relevant Provincial Health Directorate and the chief physician offices of both hospitals. Participation was entirely voluntary. All participants were informed about the study's aim, methods and data confidentiality. Informed consent was obtained from all nurses prior to data collection. To ensure anonymity, no identifying information was collected on the data forms.

## 3 Results

**FIGURE 1:**
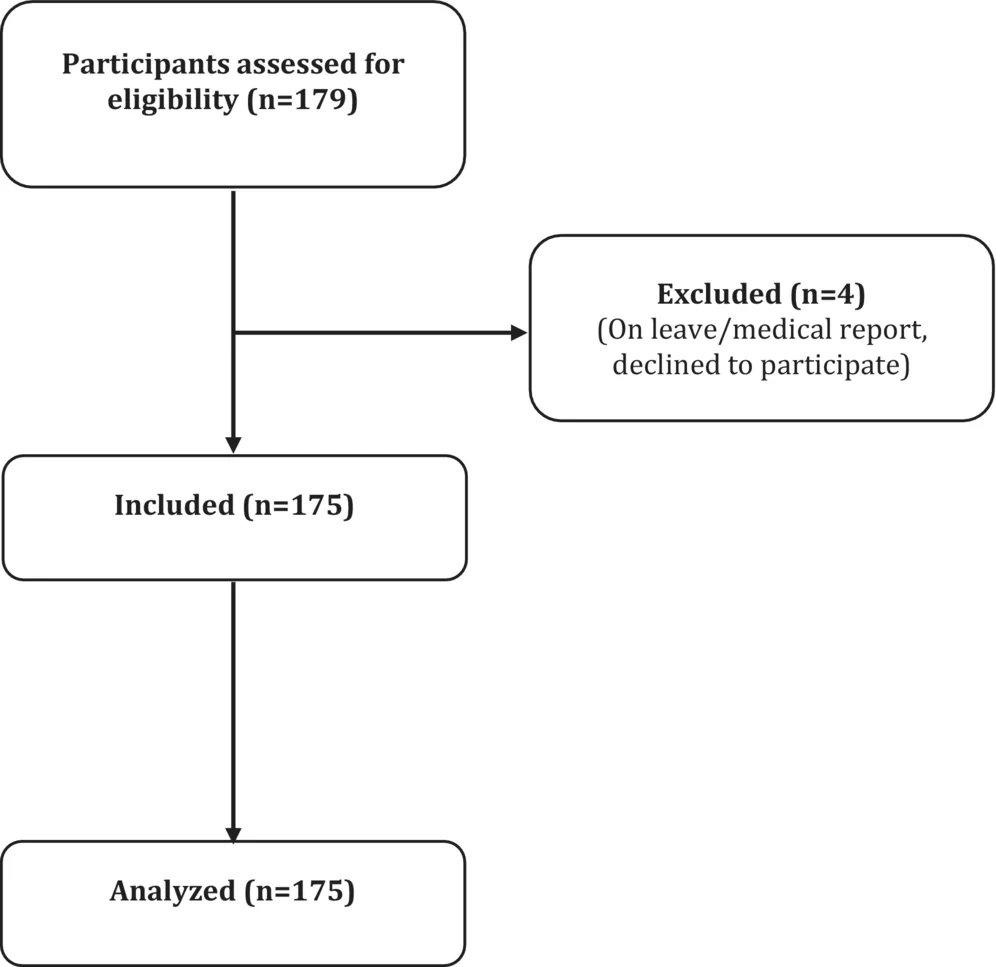
STROBE flow diagram.

Out of the 179 nurses identified in the initial population, 175 voluntarily agreed to participate and completed the questionnaires, resulting in a high response rate of 97.8%. The remaining four nurses (2.2%) were excluded from the study due to various reasons, such as being on leave, medical reports or declining to participate (Figure 1).

### 3.1 Demographic and Professional Characteristics of Nurses

All nurses were female, with a mean age of 30.07 ± 4.64 years. The majority held a bachelor's degree (70.3%) and worked in NICU (76.0%). Most had ≤ 5 years of professional experience (43.4%) and worked ≤ 180 h per month (56.6%). While nearly half were undecided about job satisfaction (48.6%), satisfaction with the working unit was notably higher (63.4%) (Table 1).

**TABLE 1:** Distribution of demographic and professional characteristics of nurses (*n* = 175).

Variables	Mean ± SD	Min.–Max.
Age (years)	30.07 ± 4.64	24–46

	Frequency (*n*)	Percentage (%)
Level of education		
High school and associate degree	14	8.0
Bachelor's degree	123	70.3
Master's degree	38	21.7
Unit		
Neonatal intensive care	133	76.0
Paediatric intensive care	42	24.0
Professional experience		
0–5 years	76	43.4
5–10 years	59	33.7
Over 10 years	40	22.9
Monthly working hours		
≤ 180 h	99	56.6
181–200 h	42	24.0
201–220 h	17	9.7
> 220 h	17	9.7
Job satisfaction		
Satisfied	63	36.0
Undecided	85	48.6
Dissatisfied	27	15.4
Unit satisfaction		
Satisfied	111	63.4
Undecided	47	26.9
Dissatisfied	17	9.7

Abbreviations: Min, minimum; Max, maximum; SD, standard deviation.

### 3.2 Descriptive Statistics of Scales

According to the findings of Part A of the MISSCARE Survey**—**Paediatric Version, protocol‐based fundamental nursing care practices such as infection control, vital signs monitoring, hand hygiene, body hygiene and catheter care were largely reported as being performed ‘always’ (over 85%). In contrast, notable deficiencies were observed in mobility‐related care practices. Assisting the child with ambulation three times per day or in accordance with the nursing care plan (66.9% ‘never’), helping with toileting needs within 5 min (66.3% ‘never’) and performing passive mobilisation every 2 h (33.7% ‘never’) were identified as the most frequently missed care activities. For time‐sensitive nursing interventions, including acting on medication requests within 15 min and responding to call lights within 5 min, the proportion of ‘always’ responses ranged between 45% and 53%. Responses related to family‐centred and developmental care practices demonstrated greater variability, with ‘always’ rates ranging from 35% to 56%. Overall, these findings provide an answer to the second research question by indicating that mobility‐related interventions were the most frequently missed nursing care activities, whereas labour‐ and communication‐related factors constituted the principal reasons for missed nursing care in neonatal and paediatric intensive care units. Additional statistical results are provided in Table S1.

The mean AFQ score was 15.46 ± 5.88, indicating that nurses experienced alarm fatigue. This finding addresses the first research question concerning the level of alarm fatigue among nurses working in neonatal and paediatric intensive care units.

In Section B of the MISSCARE Survey—Paediatric Version, the mean scores for the subdimensions were 10.23 ± 2.78 (range: 4–16) for material resources, 16.81 ± 3.79 (range: 10–28) for communication and 15.03 ± 3.18 (range: 6–20) for labour resources (Table 2). Labour and communication resource factors emerged as the most prominent reasons for missed nursing care, thereby addressing the second research question.

**TABLE 2:** Descriptive statistics for AFQ and the subdimensions of the MISSCARE Survey–Paediatric Version (Section B) (*n* = 175).

Variable	Number of items	Mean	SD	Minimum	Maximum
AFQ	9	15.46	5.88	0	29
Material resources	4	10.23	2.78	4	16
Communication	7	16.81	3.79	10	28
Labour resources	5	15.03	3.18	6	20

Abbreviations: AFQ, alarm fatigue questionnaire; SD, standard deviation.

### 3.3 Relationship Between Scale and Subdimension Scores

Significant positive correlations were identified between nurses' AFQ scores and the material (*r* = 0.268, *p* < 0.001), communication (*r* = 0.208, *p* = 0.006) and labour resource (*r* = 0.243, *p* = 0.001) subdimensions of missed nursing care (Table 3). These findings indicate that higher levels of alarm fatigue are associated with increased levels of missed nursing care, as well as greater challenges related to material, communication and labour resource factors. These findings answer the third research question and suggest that higher alarm fatigue levels are associated with greater missed nursing care‐related challenges.

**TABLE 3:** Correlations between the AFQ and the subdimensions of the MISSCARE Survey—Paediatric Version (Section B) (*n* = 175).

Variable		Material resources	Communication	Labour resources
AFQ	*r*	0.268	0.208	0.243
	*p*‐value	< 0.001	0.006*	0.001*

Abbreviations: AFQ, alarm fatigue questionnaire; *r*, Pearson's correlation coefficient.

* *p* < 0.05.

### 3.4 Regression Analysis of Alarm Fatigue and Missed Nursing Care

Simple linear regression analyses were conducted to further examine the relationship between alarm fatigue and the reasons for missed nursing care. Alarm fatigue significantly predicted material resource‐related (β = 0.267, *p* < 0.001), communication‐related (β = 0.206, *p* = 0.006) and labour resource‐related reasons for missed nursing care (β = 0.243, *p* = 0.001). However, the explained variance was limited (*R* ^2^ = 0.043–0.071), indicating that alarm fatigue accounted for a relatively small proportion of the variability in missed nursing care reasons (Table 4).

**TABLE 4:** Simple linear regression analyses examining the effect of alarm fatigue on reasons for missed nursing care (*n* = 175).

Dependent variable	B	SE	β	*t*	*p*	95% CI for B	*R* ^2^
Material resources	0.126	0.035	0.267	3.644	< 0.001	0.058–0.194	0.071
Communication	0.133	0.048	0.206	2.774	0.006	0.038–0.227	0.043
Labour resources	0.131	0.040	0.243	3.296	0.001	0.053–0.210	0.059

Abbreviations: β, standardised regression coefficient; B, unstandardized regression coefficient; CI, confidence interval; SE, standard error.

## 4 Discussion

In this study, it was determined that nurses working in the NICU and PICU performed protocol‐based and life‐sustaining care practices predominantly at the level of ‘always’. The high rates of implementation of interventions directly related to patient safety particularly infection control, assessment of vital signs and medication administration suggest that nurses prioritise clinical risk in their care prioritisation processes. Consistent with the literature, it has been reported that, in NICU and PICU settings, interventions aimed at maintaining physiological stability, such as medication administration and monitoring of vital signs, are less frequently omitted [20, 23]. A study conducted in China reported findings consistent with those of the present study, demonstrating high rates of adherence to vital signs assessment and the implementation of interventions aimed at preventing infections [29]. These findings indicate that, within the intensive care context, nurses tend to prioritise the safety dimension of care, whereas developmental and psychosocial aspects are considered of secondary importance.

In this study, care interventions related to movement and mobilisation were found to be missed more frequently than other care practices in neonatal and paediatric intensive care units. This finding is consistent with previous studies conducted in paediatric and neonatal care settings. An integrative review of 14 studies conducted across the United States, Canada, Israel and Kenya showed that missed nursing care was common in these settings. The same review identified fundamental care practices, such as oral care, patient feeding, bathing and meal preparation, as among the most frequently missed care activities [22]. Similarly, data obtained from seven states in the United States showed that care activities such as participation in daily rounds, oral care for infants receiving ventilatory support, routine bathing, facilitation of parental involvement and parental education were frequently missed in neonatal intensive care units [27]. Taken together, the present findings and previous studies suggest that care interventions requiring more time and intensive nursing workforce may be more prone to omission due to staff shortages, increased workload and prioritisation processes. In addition, the high rates of missed care observed in relation to ambulation and assistance with toileting in the present study should be interpreted within the clinical context of neonatal and paediatric intensive care units. Many patients in these settings are critically ill, mechanically ventilated or require continuous monitoring, which may limit opportunities for routine mobilisation and increase dependence on high‐priority physiological care.

In this study, the mean alarm fatigue score indicated that alarm fatigue was evident among nurses, a finding that is consistent with the existing literature. Similar findings were reported in studies conducted in Poland by Lewandowska et al. [4] and in Türkiye by Akturan et al. [30]. Furthermore, in a study conducted in the United States, Bonafide et al. [31] demonstrated that an increase in non‐actionable alarm burden prolonged nurses' response times, thereby highlighting the clinical implications of alarm fatigue. In addition, a systematic review of 14 cross‐sectional studies conducted in China, Iran and South Korea indicated that alarm fatigue among nurses was generally at a moderate level [32]. Similarly, a study conducted in the Netherlands reported that 48.7% of participating nurses experienced alarm fatigue. These differences may be attributed to variations in clinical settings, patient acuity, alarm management practices and organisational conditions.

In this study, the mean scores obtained from the reasons section of the MISSCARE Survey indicated that factors related to labour, communication and material resources were reported as relevant contributors to missed nursing care. However, when the number of items within each subdimension was considered, labour‐related factors had the highest mean score. The findings of several studies in the literature are consistent with these results. In a study conducted with paediatric nurses at a university hospital in Türkiye, Olcay and Yiğit [33] identified labour‐related factors, particularly insufficient nurse staffing, workload and working conditions, as the primary reasons for missed care, followed by material resource‐related factors. Similarly, a study conducted in paediatric emergency departments in Türkiye reported that an insufficient number of nurses and a high proportion of newly recruited nurses were among the main reasons for missed nursing care [34]. Furthermore, in a comprehensive review of paediatric nursing care, Maffeo et al. [35] emphasised that workload, work environment and staffing levels are key determinants of missed care. The prominence of labour‐related factors in this study may be associated with the structural characteristics of the intensive care environment. High patient acuity, the use of advanced technological equipment, continuous monitoring of critically ill patients and the need for rapid clinical decision‐making increase both cognitive and physical workload. This, in turn, may lead nurses to prioritise certain aspects of care, resulting in the delay or omission of some nursing interventions.

In this study, statistically significant positive correlations were identified between nurses' levels of alarm fatigue and the material‐, communication‐ and labour‐related reasons for missed nursing care. However, given the weak strength of the observed correlations, these findings should be interpreted cautiously. Although alarm fatigue appears to contribute to missed nursing care associated with labour resources, communication and material resources, it is unlikely to be the sole influencing factor. Other individual, organisational and environmental factors may also play a role in the occurrence of missed nursing care. The literature provides evidence on the impact of alarm fatigue on clinical processes. Previous research has demonstrated the potential effects of alarm burden on clinical processes. In an observational study conducted in the United States, Bonafide et al. [31] found that greater exposure to non‐actionable alarms was associated with longer nurse response times. Similarly, a study conducted in South Korea reported that frequent false alarms were perceived as a major obstacle to alarm management and could reduce nurses' attention or responses to alarms [36]. In a study conducted in the United States, Kalisch and Williams [26] identified labour resources, material resources and communication as the principal dimensions of the reasons for missed nursing care. Similarly, Ball et al. [37] found that staffing and contextual factors were associated with care left undone in a study conducted in Sweden. From the perspective of paediatric care settings, the clinical significance of this relationship becomes even more pronounced. The high monitoring demands of paediatric patients, their limited physiological tolerance and the family‐centred nature of care suggest that the increased cognitive burden associated with alarm fatigue may adversely affect not only technical monitoring processes but also core components of care such as communication, education and emotional support. At the same time, alarm fatigue among nurses may be a consequence of organisational and systemic conditions. A qualitative study conducted in Southwest China [38], a meta‐synthesis of qualitative studies from Iran, Spain, China, Türkiye, the United States and Germany [16], and qualitative research conducted in Iran identified factors such as frequent or false alarms, competing tasks, staffing shortages and inadequate organisational resources as contributors to alarm fatigue [39]. Therefore, alarm fatigue should not be viewed solely not only as an individual‐level challenge but also as a potential indicator of system‐level deficiencies that adversely affect care delivery. Furthermore, given the cross‐sectional design of the present study, the directionality of the observed associations cannot be determined.

## 5 Limitations

This study has several limitations. First, the cross‐sectional design precludes any conclusions regarding causal relationships between alarm fatigue and missed nursing care. As the data were collected through self‐reported questionnaires completed by nurses working in the NICU and PICU of two institutions, the findings cannot be generalised to all nurses in this field. All participants were female, which may limit the generalisability of the findings to male nurses working in neonatal and paediatric intensive care settings. Furthermore, although the questionnaires were completed anonymously and self‐administered by the participants, data collection was conducted through face‐to‐face visits to the units. Therefore, the results may be subject to self‐report bias and the potential influence of social desirability bias, which may have affected participants' responses regarding alarm fatigue and missed nursing care. In addition, alarm fatigue was assessed solely through self‐reported measures, and objective indicators such as alarm frequency, alarm exposure and alarm response data were not evaluated. Finally, institutional and organisational factors, including unit culture and alarm management practices, were not examined and may have influenced the findings.

## 6 Implications for Practice

We suggest that reducing alarm fatigue in NICU and PICU settings requires a comprehensive approach that includes optimising alarm management practices, improving workforce planning and ensuring balanced workload distribution. The implementation of structured alarm governance protocols, regular review of alarm settings and optimisation of patient‐specific alarm parameters may help reduce non‐actionable alarms and support timely clinical responses. Where feasible, the use of smart alarm technologies and advanced monitoring systems may further contribute to reducing unnecessary alarm exposure. Strengthening structured communication models and multidisciplinary team collaboration may also contribute to reducing missed nursing care.

In addition, ongoing educational initiatives should be prioritised. In‐service education programmes and simulation‐based alarm management training may enhance nurses' ability to recognise, prioritise and appropriately respond to alarms in complex clinical environments, thereby supporting patient safety and care quality.

We highlight the need for further studies conducted in diverse clinical settings with larger samples and qualitative designs to better understand the relationship between alarm fatigue and missed nursing care.

## 7 Conclusions

This study found that nurses reported alarm fatigue and that higher alarm fatigue levels were associated with increased reasons for missed nursing care. The findings suggest that alarm fatigue may be related to organisational and care delivery challenges in NICU and PICU settings. Although the observed associations were statistically significant, their magnitude was weak and should therefore be interpreted with caution. Nevertheless, these findings highlight the potential importance of alarm management practices, workforce capacity and organisational factors in supporting the continuity and quality of nursing care and promoting patient safety.

## Funding

The authors have nothing to report.

## Ethics Statement

The study was conducted in accordance with the Declaration of Helsinki. Ethics approval was obtained from the Sakarya Applied Sciences University Ethics Committee (Date: 7 May 2025; No: E‐26428519‐050.99‐172 770). Institutional permissions were secured from the relevant Provincial Health Directorate and the chief physician offices of both hospitals.

## Consent

Informed consent was obtained from all participants included in the study.

## Conflicts of Interest

The authors declare no conflicts of interest.

## Supporting information


**Table S1:** Percentage and frequency distribution of nurses' responses to Part A of the MISSCARE Survey—Pa**e**diatric Version (*n* = 175).

## Data Availability

The data that support the findings of this study are available on request from the corresponding author. The data are not publicly available due to privacy or ethical restrictions.
