# Supplementary material for: Alarm Fatigue and Missed Nursing Care Among Neonatal and Paediatric Intensive Care Nurses: A Cross‐Sectional Study
Source: Nurs Crit Care. 2026 Aug 24;31(5):e70649. doi: 10.1111/nicc.70649 (PMC13504183; doi:10.1111/nicc.70649)
Supplement: Supplementary file 1 [file NICC-31-0-s001.docx]

**SUPPLEMENTARY TABLE.** Percentage and frequency distribution of nurses’ responses to Part A of the MISSCARE Survey–Paediatric Version (n = 175).

|  | Never | | Rarely | | Sometimes | | Often | | Always | |
| --- | --- | --- | --- | --- | --- | --- | --- | --- | --- | --- |
|  | **n** | **%** | **n** | **%** | **n** | **%** | **n** | **%** | **n** | **%** |
| 1. Attendance at daily rounds at the bedside | 0 | 0,0 | 0 | 0,0 | 0 | 0,0 | 21 | 12,0 | 154 | 88,0 |
| 2. Ambulation 3 times per day or as per nursing plan, if clinical conditions allows | 117 | 66,9 | 14 | 8,0 | 7 | 4,0 | 19 | 10,9 | 18 | 10,3 |
| 3. Assess effectiveness of medications | 1 | 0,6 | 4 | 2,3 | 20 | 11,4 | 51 | 29,1 | 99 | 56,6 |
| 4. Passive mobilization of child every 2 h or as ordered | 59 | 33,7 | 10 | 5,7 | 5 | 2,9 | 35 | 20,0 | 66 | 37,7 |
| 5. Mouth care | 0 | 0,0 | 0 | 0,0 | 12 | 6,9 | 58 | 33,1 | 105 | 60,0 |
| 6. Parents included in child's care | 5 | 2,9 | 16 | 9,1 | 42 | 24,0 | 51 | 29,1 | 61 | 34,9 |
| 7. Patient and family education | 5 | 2,9 | 0 | 0,0 | 30 | 17,1 | 42 | 24,0 | 98 | 56,0 |
| 8. Discussion with child and his/her family about plans for discharge and care at home | 5 | 2,9 | 4 | 2,3 | 21 | 12,0 | 50 | 28,6 | 95 | 54,3 |
| 9. Promote neuro-evolutionary develop, according to age and baby's clinical conditions (i.e., neonatal care, cognitive and relational development in child or in adolescent) | 29 | 16,6 | 10 | 5,7 | 26 | 14,9 | 39 | 22,3 | 71 | 40,6 |
| 10. Pain assessment with pharmacological or with nonpharmacological care approaches, according to protocol | 4 | 2,3 | 3 | 1,7 | 16 | 9,1 | 60 | 34,3 | 92 | 52,6 |
| 11. Medication requests acted on in 15 min | 18 | 10,3 | 8 | 4,6 | 22 | 12,6 | 49 | 28,0 | 78 | 44,6 |
| 12. Full documentation of all necessary data | 1 | 0,6 | 1 | 0,6 | 7 | 4,0 | 33 | 18,9 | 133 | 76,0 |
| 13. Communication of all relevant information during shift change or handover | 2 | 1,1 | 0 | 0,0 | 0 | 0,0 | 28 | 16,0 | 145 | 82,9 |
| 14. Satisfaction of eating need according to child's clinical conditions (i.e., encourages oral feeding and/or nutrition at the request on the newborn; encourage a correct alimentation in according of personal taste) | 0 | 0,0 | 1 | 0,6 | 0 | 0,0 | 17 | 9,7 | 157 | 89,7 |

| 15. Medications administered in 30 min before or after scheduled time (i.e., scheduled time 8 p.m., administration between 7:30 p.m. and 8:30 p.m.) | 13 | 7,4 | 23 | 13,1 | 38 | 21,7 | 24 | 13,7 | 77 | 44,0 |
| --- | --- | --- | --- | --- | --- | --- | --- | --- | --- | --- |
| 16. Assist child with toileting needs in 5 min of request (i.e., go with baby to the toilet or give appropriate devices if bedridden) | 116 | 66,3 | 2 | 1,1 | 1 | 0,6 | 28 | 16,0 | 28 | 16,0 |
| 17. Response to call light, to intervention request, or alarm is initiated in 5 min (i.e., monitor, infusion pumps, ventilator) | 44 | 25,1 | 2 | 1,1 | 4 | 2,3 | 33 | 18,9 | 92 | 52,6 |
| 18. Emotional support to child and/or family | 9 | 5,1 | 8 | 4,6 | 30 | 17,1 | 48 | 27,4 | 80 | 45,7 |
| 19. Labs/specimen obtained as ordered | 4 | 2,3 | 0 | 0,0 | 0 | 0,0 | 16 | 9,1 | 155 | 88,6 |
| 20. Body hygiene and skin care | 0 | 0,0 | 0 | 0,0 | 0 | 0,0 | 12 | 6,9 | 163 | 93,1 |
| 21. Central line site and peripheral line site assessment per protocol | 0 | 0,0 | 0 | 0,0 | 4 | 2,3 | 19 | 10,9 | 152 | 86,9 |
| 22. Central line site and peripheral line site care per protocol | 0 | 0,0 | 4 | 2,3 | 2 | 1,1 | 19 | 10,9 | 150 | 85,7 |
| 23. Adoption of the necessary precautions for infections control as per protocol (Individual Protection Devices, devices disinfection, isolation, correct waste disposal) | 0 | 0,0 | 0 | 0,0 | 2 | 1,1 | 15 | 8,6 | 158 | 90,3 |
| 24. Monitoring intake/output of solid and liquid | 5 | 2,9 | 0 | 0,0 | 15 | 8,6 | 20 | 11,4 | 135 | 77,1 |
| 25. Vital signs assessed according to the nursing plan | 0 | 0,0 | 0 | 0,0 | 1 | 0,6 | 10 | 5,7 | 164 | 93,7 |
| 26. Focused reassessments of the child's condition to assess improvements or deterioration during the shift | 2 | 1,1 | 0 | 0,0 | 1 | 0,6 | 32 | 18,3 | 140 | 80,0 |
| 27. Hand washing | 0 | 0,0 | 1 | 0,6 | 0 | 0,0 | 11 | 6,3 | 163 | 93,1 |
| 28. Assessment of the activities attributed to caregiver | 24 | 13,7 | 6 | 3,4 | 6 | 3,4 | 18 | 10,3 | 121 | 69,1 |
| 29. Safety and hygiene checks of bedside equipment completed once per shift or per protocol (i.e., bed, nightstand, devices) | 3 | 1,7 | 3 | 1,7 | 5 | 2,9 | 20 | 11,4 | 144 | 82,3 |
